# Beyond dynamical mean-field theory of neural networks

**DOI:** 10.1186/1471-2202-14-S1-P60

**Published:** 2013-07-08

**Authors:** Massimiliano Muratori, Bruno Cessac

**Affiliations:** 1 NeuroMathComp team (INRIA, UNSA LJAD), Sophia Antipolis, France

## 

**Figure 1:**
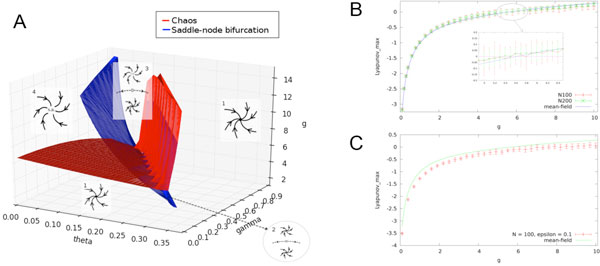
**(A) Bifurcation map**. 1 : one stable fixed point; 2 two stable fixed points; 3 one fixed point and one strange attractor; 4 one strange attractor. (B) Finite N and Mean-Field Maximal Lyapunov exponent (θ = 0.1, γ = 0). (C) Finite N Maximal Lyapunov exponent with weak correlation (ε = 0.01 and Mean-Field Maximal Lyapunov Exponent without correlation (ε = 0).

We consider a set of N firing rate neurons with discrete time dynamics and a leak term γ. The nonlinearity of the sigmoid is controlled by a parameter g and each neuron has a firing threshold θ, Gaussian distributed (thresholds are uncorrelated). The network is fully connected with correlated Gaussian random synaptic weights, with mean zero and covariance matrix C/N. When synaptic weights are uncorrelated the dynamic mean field theory developed in [1-3] allows us to draw the bifurcation diagram of the model in the thermodynamic limit (N tending to infinity): in particular there is sharp transition from fixed point to chaos characterized by the maximum Lyapunov exponent, which is known analytically in the thermodynamic limit. The bifurcation diagram is drawn in Figure 1 A. However, mean-field theory is exact only in the thermodynamic limit and when synaptic weights are uncorrelated. What are the deviations from mean-field theory observed when one departs from these hypotheses? We have first studied the finite size dynamics. For finite N the maximal Lyapunov exponent has a plateau at 0 corresponding to a transition to chaos by quasi-periodicity where dynamics is at the edge of chaos (Figure 1 B). This plateau disappears in the thermodynamic limit. Thus, mean-field theory neglects an important finite-sized effect since neuronal dynamics at the edge of chaos has strong implications on learning performances of the network [4]. We also studied the effect of a weak correlation (of amplitude ε) on dynamics. Even, when ε is small one detects an important deviation on the maximal Lyapunov exponent (Figure 1 C).
